# Partial recovery of tuberculosis preventive treatment in Brazil after pandemic drawback

**DOI:** 10.1590/0102-311XEN192923

**Published:** 2024-05-20

**Authors:** Iane Coutinho, Layana Costa Alves, Guilherme Loureiro Werneck, Anete Trajman

**Affiliations:** 1 Universidade do Estado do Rio de Janeiro, Rio de Janeiro, Brasil.; 2 Universidade Federal da Bahia, Salvador, Brasil.; 3 Universidade Federal do Rio de Janeiro, Rio de Janeiro, Brasil.; 4 McGill University, Montreal, Canada.

**Keywords:** SARS-CoV-2, Coronavirus, Tuberculosis, Isoniazid, Rifampin, SARS-CoV-2, Coronavirus, Tuberculose, Isoniazida, Rifampina, SARS-CoV-2, Coronavirus, Tuberculosis, Isoniazida, Rifampin

## Abstract

Brazil was heavily affected by COVID-19 both with death toll and economically, with absence of a centralized Federal Government response. Tuberculosis (TB) notifications decreased in 2020 but partial recovery was observed in 2021. We have previously shown a sharp (93%) reduction in TB preventive treatment notifications among five Brazilian cities with more than 1,000 notifications in 2021. We hypothesized TB preventive treatment would also recover. We updated the previous analysis by adding other cities that hold more than a 1,000 notifications until 2022. Data aggregated by 2-week periods were extracted from the Information System for Notifying People Undergoing Treatment for LTBI (IL-TB). Biweekly percentage change (BPC) of notifications until October 2022 and outcomes until July 2022 (in the two weeks of TB preventive treatment initiation) were analyzed using Joinpoint software. A total of 39,701 notifications in 11 cities were included, 66% from São Paulo and Rio de Janeiro, Brazil. We found a significant increase of TB preventive treatment notifications in the beginning of 2021 (BPC range 1.4-49.6), with sustained progression in seven out of the 11 cities. Overall, median completion rates were 65%. In most cities, a gradual and steady decrease of treatment completion rates was found, except for Rio de Janeiro and Manaus (Amazonas State, Brazil), where a BPC of 1.5 and 1.2, respectively, was followed by a sustained increase. Notifications and completion proportions of TB preventive treatment were heterogeneous, which partly reflects the heterogeneity in local response to the pandemic. We found that notifications were recovered, and that the sharp 2021 decrease was no longer observed, which suggests delays in notification. In conclusion, the sharp reductions in TB preventive treatment completion rates in most cities might have been caused by delays in reporting; however, the sustained and progressive decrease are a concern.

## Resumo

O Brasil foi fortemente atingido pela COVID-19 tanto com número de mortes quanto economicamente, com ausência de uma resposta centralizada do Governo Federal. As notificações de tuberculose (TB) diminuíram em 2020, mas se recuperaram parcialmente em 2021. Já mostramos uma redução acentuada (93%) nas notificações de tratamento preventivo de TB nas cinco cidades brasileiras com mais de 1.000 notificações em 2021. Hipotetizamos que o tratamento preventivo de TB também recuperar-se-ia. Atualizamos a análise anterior acrescentando outras cidades que apresentaram mais de 1.000 notificações até 2022. Os dados agregados por períodos de duas semanas foram extraídos do Sistema de Informação para Notificação das Pessoas em Tratamento de ILTB (IL-TB). As notificações quinzenais de variação percentual até outubro de 2022 e os desfechos até julho de 2022 (nas duas semanas de início do tratamento precoce de TB) foram analisados usando o software Joinpoint. Foram incluídas 39.701 notificações em 11 cidades, sendo 66% delas de São Paulo e do Rio de Janeiro (Brasil). Encontramos um aumento significativo das notificações de tratamento preventivo de TB no início de 2021 (faixa de variação quinzenal percentual 1,4-49,6), com progressão sustentada em 7/11 cidades. No geral, as taxas medianas de conclusão foram de 65%. Na maioria dos municípios, houve queda gradual e constante das taxas de conclusão de tratamento, com exceção do Rio de Janeiro e Manaus (Amazonas, Brasil), onde a variação quinzenal percentual de 1,5 e 1,2, respectivamente, foi acompanhada de aumento sustentado. As notificações e proporções de tratamento preventivo de TB completados foram heterogêneas, o que reflete em parte a diversidade na resposta local à pandemia. No geral, as notificações se recuperaram e a queda acentuada de 2021 não é mais observada, o que sugere atrasos na notificação. Em conclusão, a redução das taxas de conclusão do tratamento preventivo da TB na maioria das cidades pode refletir atrasos na notificação, mas a diminuição sustentada e progressiva das notificações preocupa.

## Resumen

Brasil fue seriamente afectado por el COVID-19, tanto con el número de muertes como económicamente, con la ausencia de una respuesta centralizada del Gobierno Federal. Las notificaciones de la tuberculosis (TB) redujeron en 2020, pero aumentaron parcialmente en 2021. Ya mostramos una reducción drástica (el 93%) en las notificaciones del tratamiento preventivo de la TB en las cinco ciudades brasileñas con más de 1.000 notificaciones en 2021. Nuestra hipótesis es que el tratamiento preventivo de la TB también aumentaría. Actualizamos el análisis anterior añadiendo otras ciudades que presentaron más de 1.000 notificaciones hasta 2022. Los datos agregados durante períodos de dos semanas se extrajeron del Sistema de Información de Notificaciones para Personas en Tratamiento por ILTB (IL-TB). Las notificaciones quincenales de cambio porcentual hasta octubre de 2022 y os resultados hasta julio de 2022 (en las dos semanas iniciales del tratamiento precoz de la tuberculosis) se analizaron a través del software Joinpoint. Se incluyeron 39.701 notificaciones en 11 ciudades, siendo el 66% de ellas en São Paulo y Rio de Janeiro, Brasil. Encontramos un aumento significativo de las notificaciones del tratamiento preventivo de la TB a principios de 2021 (rango de cambio porcentual quincenal 1,4-49,6), con progresión sostenida en siete de las once ciudades. En general, las tasas medias de finalización fueron del 65%. En la mayoría de los municipios, hubo una reducción gradual y constante de las tasas de finalización de tratamiento, salvo en Rio de Janeiro y Manaus (Amazonas, Brasil), donde el cambio porcentual quincenal de 1,5 y 1,2, respectivamente, estuvo acompañado de un aumento sostenido. Las notificaciones y proporciones de cumplimentación del tratamiento preventivo de la TB fueron heterogéneas, lo que refleja la heterogeneidad en la respuesta local a la pandemia. En general, las notificaciones aumentaron y ya no se observa la fuerte caída de 2021 lo que refleja en parte retrasos en la notificación. En conclusión, la reducción en las tasas de finalización del tratamiento preventivo de la TB en la mayoría de las ciudades puede reflejar retrasos en la notificación, pero la reducción sostenida y progresiva es una preocupación.

## Introduction

Tuberculosis (TB) preventive treatment is one of the main strategies for reducing TB incidence ^1^ ^,^ ^2^. The United Nations’ High-level Meetings committed to offer 30 million TB preventive treatment from 2018 to 2022 ^3^, and 45 million from 2023 to 2027 ^4^. To improve TB preventive treatment, in recent years, the Brazilian Ministry of Health has implemented a TB preventive treatment information system, the Informations System for Notifying People Undergoing Treatment for LTBI (IL-TB, http://sitetb.saude.gov.br/iltb/login.seam), adopted interferon-gamma release assays for TB infection diagnosis in high-risk populations ^5^, and a shorter and safer regimen as the first choice for TB preventive treatment (3HP: three months of weekly oral doses of rifapentine & isoniazid) ^6^ ^,^ ^7^. The Brazilian Ministry of Health is also supporting the ExpandTPT (TBREACH Stop TB partnership 10429) project, which aims to improve TB preventive treatment by training and qualification of healthcare professionals in five cities with high TB burden ^(^ ^8^.

The COVID-19 pandemic caused a severe drawback in the ongoing advances for TB elimination in 2020, with a substantial drop in TB detection ^9^. Partial recovery was observed in 2021 in many countries, including Brazil ^10^. The impact of the pandemic in TB prevention was less explored. We have previously reported a 93% drop of notifications in 2020 and in the first semester of 2021 in five Brazilian cities that had notified at least 1,000 TB preventive treatment ^11^. The analyses were updated to verify to which extent this notification decrease was due to delays.

## Methods

### Study design

This is an update of a previously published retrospective cohort study based on secondary data ^11^. Methods were the same as reported previously, summarized as follows.

### Setting

TB preventive treatment is recommended in Brazil for individuals of all ages who had contacts with TB infection, people living with HIV (PLHIV) and other high-risk populations ^1^. In 2018, a digital surveillance information system for latent tuberculosis infection was implemented, the IL-TB ^12^, and the number of TB preventive treatment notifications increased steadily until the COVID-19 pandemic ^13^ ^,^ ^14^, dropping by 93% in the five capitals that had notified at least 1,000 TB preventive treatment up to July 2021 ^11^. Available regimens are 6 or 9 months of daily isoniazid, four months of daily rifampicin ^12^, and, more recently, 12 weekly doses of rifapentine plus isoniazid (3HP), since August 2021 ^6^ ^,^ ^7^.

### Eligibility

All cities with at least 1,000 notifications until October 2022 were included.

### Outcomes

The number of TB preventive treatment notified and the proportion of different treatment outcomes were evaluated according to the week of prescription - outcome cohort. The following treatment outcomes are available in the IL-TB system: treatment completed, loss to follow-up, death, transfer out, active TB during preventive treatment, adverse events resulting in treatment interruption, suspended due to unfavourable clinical condition, and suspended after a negative tuberculin skin test (TST) (< 5mm) in newborns started on TB preventive treatment before testing.

### Study period

The database contains TB preventive treatment notifications from October 2018 to October 2022. This study considers outcomes from October 2018 to July 2022.

### Data source and variables

The anonymized data was aggregated by 2-week periods and were extracted from the IL-TB from the Brazilian Ministry of Health. The following variables were used: city of initial treatment, regimen prescribed (isoniazid, rifampicin, and rifapentine associated with isoniazid), date of prescription, date of treatment completion/interruption, treatment outcome, number of doses dispensed (for the notifications with outcome informed), and record date of treatment outcome.

### Analysis

For missing outcomes, two different outcomes were attributed: probable loss to follow-up (more than 120% of regimen length plus 90 days to allow for notification delay) or possibly still under treatment (all others). Outcomes classified as possibly under treatment were excluded from the outcome analyses. Outcomes classified as probable loss to follow-up were considered as such.

The biweekly data were analyzed in RStudio version 4.2.1 (https://rstudio.com/) by city. Notifications and outcomes were presented as absolute numbers and proportion over TB preventive treatment notifications, respectively.

The Shapiro-Wilk test was used for testing normality. For normally distributed data, the t-test was used to analyze overall changes over time. For non-normally distributed data, the Wilcoxon test was used. The Joinpoint software (https://surveillance.cancer.gov/joinpoint/) was used to estimate biweekly percentage change (BPC).

## Results

**Figure 1:**
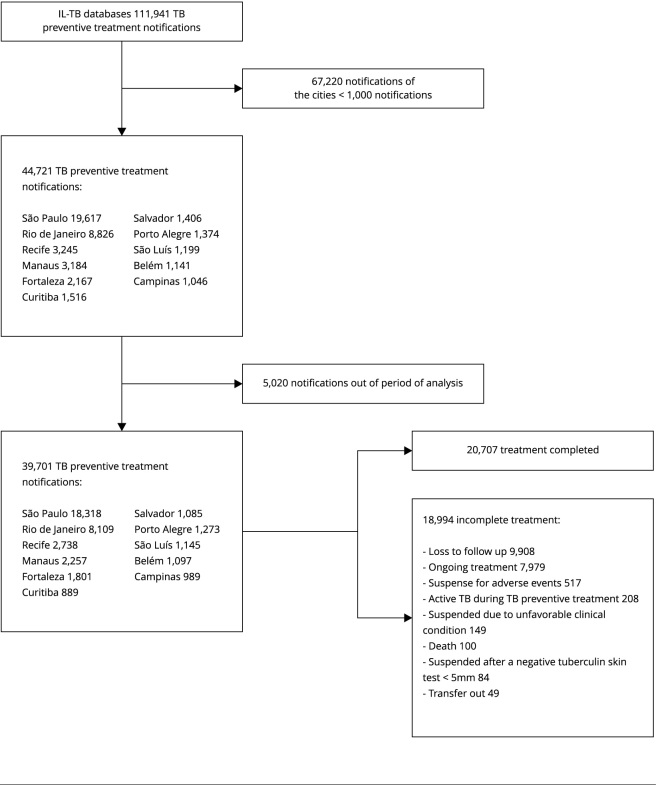
Flowchart diagram of included records.

IL-TB: Information System for Notifying People Undergoing Treatment for LTBI; TB: tuberculosis.

The database contained 111,941 notifications from 2,111 cities in 25 states, of which 11 presented more than 1,000 notifications (Figure 1): São Paulo, Rio de Janeiro, Recife (Pernambuco State), Manaus (Amazonas State), Fortaleza (Ceará State), Curitiba (Paraná State), Salvador (Bahia State), Porto Alegre (Rio Grande do Sul State), São Luís (Maranhão State), Belém (Pará State), and Campinas (São Paulo State), totaling 39,701 notifications (35% of the total notifications). São Paulo (18,318) and Rio de Janeiro (8,109) accounted for 66% of the total notifications.

Most cases occurred in women (53%) with a mean age of 36 years, 60% were self-declared black or mixed-race, 20% were PLHIV, 39% were not evaluated for HIV, 61% were contacts to TB cases, and 74% were immunized with Bacillus Calmette-Guérin vaccine (BCG).

### TB preventive treatment notifications

Monotherapy with isoniazid regimens were prescribed in 87% of notifications, ranging from 56% (Manaus) to 95% (São Luís), with a mean treatment time of 188 days. 3HP was the second-choice regimen (9%), ranging from 1% (Campinas) to 33% (Manaus). Across the other cities, the 3HP proportions were: 2% in Recife, 3% in São Paulo, 4% in São Luís, 4% in Curitiba, 7% in Salvador, 9% in Porto Alegre, 14% in Fortaleza, 16% in Rio de Janeiro, and 21% in Belém. The mean number of notifications by epidemiological week (EW) increased over the analyzed period, with a substantial increase from 2019 to 2020 (from 123 to 185; 51%; p < 0,001) and from 2021 to the EW 40, 2022 (from 203 to 296; 45%; p < 0.001) (Table 1).

**Table 1:** Joinpoint of notified tuberculosis (TB) preventive treatment and of proportion of outcomes.

EW (year)	BPC	95%CI	Prob > |t|
São Paulo			
Notified TB preventive treatment			
47 (2018) to 5 (2019)	46.4 *	26.7; 69.2	< 0.001
5 (2019) to 41 (2019)	4.5 *	2.1; 6.9	< 0.001
41 (2019) to 15 (2020)	-3.6	-7.3; 0.1	0.057
15 (2020) to 29 (2021)	1.4 *	0.6; 2.3	0.001
29 (2021) to 40 (2022)	1.8 *	0.7; 2.9	0.002
Proportion of outcomes			
1 (2019) to 41 (2021)	-0.3 *	-0.4; -0.2	< 0.001
41 (2021) to 1 (2022)	3.0	-1.9; 8.2	0.229
1 to 30 (2022)	-1.4 *	-2.4; -0.4	0.008
Rio de Janeiro			
Notified TB preventive treatment			
41 (2018) to 11 (2020)	1.7 *	1.3; 2.1	< 0.001
11 (2020) to 40 (2022)	1.8 *	1.0; 2.7	< 0.001
Proportion of outcomes			
1 (2019) to 23 (2021)	-0.5 *	-0.7; -0.3	< 0.001
23 (2021) to 30 (2022)	1.5 *	0.9; 2.1	< 0.001
Recife			
Notified TB preventive treatment			
17 (2019) to 9 (2020)	-0.5	-2.6; 1.7	0.668
9 (2020) to 17 (2020)	-27.1	-53.5; 14.2	0.163
17 (2020) to 31 (2021)	4.6 *	3.3; 5.9	< 0.001
31 (2021) to 40 (2022)	-0.7	-2.4; 1.1	0.450
Proportion of outcomes			
17 (2019) to 19 (2022)	-1.0 *	-1.7; -0.2	0.015
19 to 30 (2022)	-53.4 *	-71.5; -23.6	0.003
Manaus			
Notified TB preventive treatment			
39 (2019) to 19 (2020)	-8.2 *	-11.8; -4.5	< 0.001
19 (2020) to 47 (2020)	6.2 *	0.6; 12.2	0.032
47 (2020) to 1 (2021)	-44.6	-80.6; 57.6	0.257
1 (2021) to 13 (2021)	49.6 *	18.4; 89.0	0.001
13 (2021) to 40 (2022)	1.9 *	1.0; 2.9	< 0.001
Proportion of outcomes			
39 (2019) to 19 (2022)	-0.1	-0.2; 0.1	0.605
19 to 30 (2022)	1.2 *	0.2; 2.1	0.015
Fortaleza			
Notified TB preventive treatment			
29 (2019) to 7 (2020)	-0.2	-4.4; 4.1	0.907
7 to 19 (2020)	-20.1	-36.2; 0.1	0.051
19 to 33 (2020)	16.3	-1.9; 37.9	0.080
33 (2020) to 3 (2022)	-0.7	-2.2; 0.8	0.342
3 to 40 (2022)	4.0 *	1.4; 6.7	0.004
Proportion of outcomes			
29 (2019) to 30 (2022)	-1.0 *	-1.8; -0.2	0.017
Porto Alegre			
Notified TB preventive treatment			
41 (2019) to 5 (2020)	29.5 *	10.9; 51.2	0.002
5 (2020) to 3 (2022)	0.2	-0.8; 1.2	0.675
3 (2022) to 40 (2022)	1.8	-1.7; 5.3	0.297
Proportion of outcomes			
35 (2019) to 30 (2022)	-0.1	-0.3; 0.2	0.721
São Luís			
Notified TB preventive treatment			
1 to 9 (2020)	10.4	-28.4; 70.0	0.647
9 to 15 (2020)	-58.8	-89.5; 61.7	0.198
15 to 31 (2020)	32.1 *	10.1; 58.6	0.004
31 (2020) to 47 (2021)	0.1	-1.5; 1.8	0.878
47 (2021) to 40 (2022)	0.9	-1.8; 3.8	0.489
Proportion of outcomes			
1 (2020) to 30 (2022)	-0.2	-0.4; 0.0	0.068
Salvador			
Notified TB preventive treatment			
43 (2019) to 15 (2020)	-1.3	-8.0; 5.9	0.711
15 to 21 (2020)	-45.5	-83.4; 78.7	0.311
21 (2020) to 29 (2020)	65.3	-8.7; 199.5	0.095
29 (2020) to 23 (2022)	0.5	-0.4; 1.3	0.287
23 to 31 (2022)	7.2 *	6.7; 7.7	< 0.001
31 to 40 (2022)	5.7 *	5.4; 6.0	< 0.001
Proportion of outcomes			
1 (2019) to 30 (2022)	-0.1	-0.7; 0.4	0.69
Belém			
Notified TB preventive treatment			
1 to 11 (2019)	34.3	-1.0; 82.1	0.058
11 (2019) to 11 (2020)	-1.3	-3.9; 1.3	0.314
11 (2020) to 21 (2020)	-28.5	-53.5; 10.1	0.124
21 (2020) to 49 (2020)	17.0 *	9.0; 25.7	< 0.001
49 (2020) to 33 (2022)	2.6 *	1.6; 3.6	< 0.001
Proportion of outcomes			
1 (2019) to 49 (2021)	-0.7	-1.3; 0.0	0.062
49 (2021) to 30 (2022)	-24.1 *	-29.3; -18.4	< 0.001
Curitiba			
Notified TB preventive treatment			
37 (2019) to 25 (2020)	-10.5 *	-14.5; -6.3	< 0.001
25 (2020) to 32 (2021)	3.5 *	0.9; 6.3	0.010
32 (2021) to 40 (2022)	2.7*	0.7; 4.8	0.010
Proportion of outcomes			
35 (2019) to 30 (2022)	-0.8	-1.8; 0.3	0.145
Campinas			
Notified TB preventive treatment			
1 to 19 (2019)	30.7 *	9.9; 55.4	0.003
19 (2019) to 49 (2020)	-2.2 *	-3.9; -0.4	0.020
49 (2020) to 40 (2022)	1.9 *	1.1; 2.8	< 0.001
Proportion of outcomes			
1 (2019) to 19 (2022)	-0.1	-0.4; 0.1	0.283
19 to 25 (2022)	-77.2 *	-89.8; -49.0	< 0.001
25 to 30 (2022)	12.7	-49.7; 152.1	0.769

95%CI: 95% confidence interval; BPC: biweekly porcentage change; EW: epidemiological week.

* p-value < 0.05.

**Figure 2:**
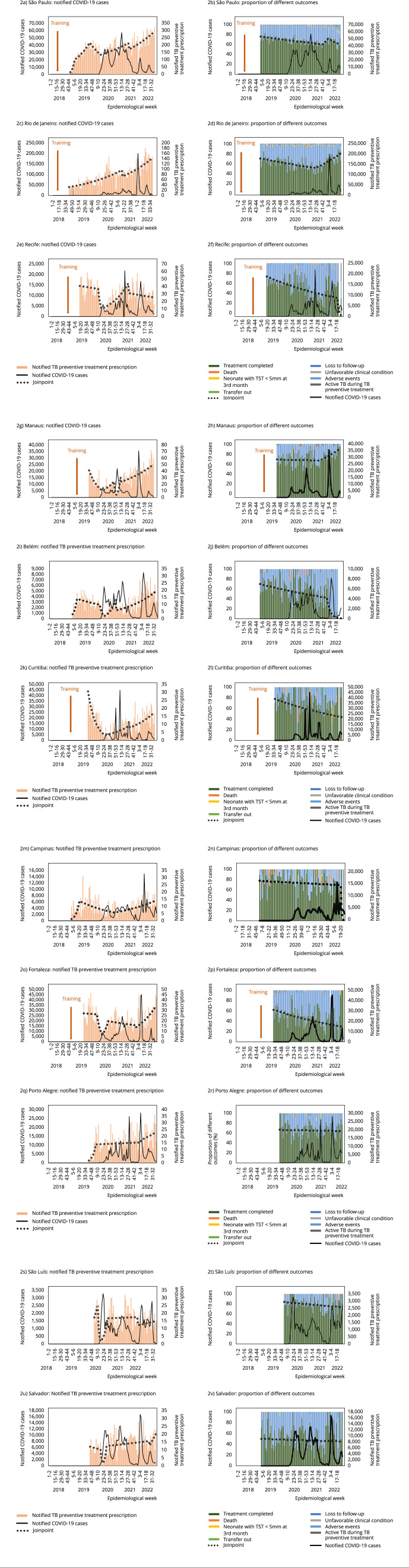
Number of notified tuberculosis (TB) preventive treatment (pink bars on the left), proportion of TB preventive treatment outcomes by date of TB preventive treatment initiation (colored bars on the right), and number of COVID-19 cases reported (black line) during the pandemic in 11 Brazilian capitals.

TB: tuberculosis; TST: tuberculin skin test. Note: treatment outcome shown on weeks of TB preventive treatment prescription. For calculation of proportions, possibly ongoing treatments were excluded from the denominator.

The increase of TB preventive treatment notifications was observed in seven of the 11 cities (BPC range = 1.4-49.6), as seen in Figures 2a to 2n. Table 1 shows a nonsignificant increase in three cities, as well as a nonsignificant decrease in one city. Manaus held the highest BPC in 2021 (EW 1, 2021 to EW 13, 2021; BPC = 49.6; Table 1). São Paulo and Rio de Janeiro presented a more progressive notification increase over time (Figure 2). In all eight cities, the progress was sustained after the inflexion. Salvador and Fortaleza showed later recoveries in 2022 (EW 23 to 31, BPC = 7.2 and EW 3 to 40, BPC = 4.0, respectively) (Table 1 and Figure 2).

### Proportion of outcomes

In total, 11,489 (29%) missing outcomes were obtained, of which 3,510 were classified as loss to follow-up and 7,979 as ongoing treatment, thus excluded from the outcome analysis.

Overall completion proportion was 65%; 31% were lost to follow-up. From 2019 to 2021, a significant decrease in the proportion of treatment completion was found.

São Luís, Manaus, and Campinas were the cities with the highest treatment completion rates (66.0%, 61.9%, and 61.7% respectively). In most cities, a non-significant gradual and steady decline was seen in treatment completion rates, except in Rio de Janeiro and Manaus, which presented a BPC of 1.5 and 1.2, respectively, followed by a sustained increase (Table 1 and Figure 2).

## Discussion

In this updated analysis, we observed that the sharp 93% reduction of TB preventive treatment notification previously reported ^11^ was actually due to notification delays. The reduction in procedures did not occur; most cities presented a transient decrease followed by a rapid recovery of TB preventive treatment prescriptions, with a sustained progression. This corroborates what has been reported regarding TB detection in the country ^10^. However, TB preventive treatment notification trends were heterogeneous across cities, evinced by the difference in the proportions of the regimens by city, possibly reflecting heterogeneity in the local response to the pandemic.

The recovery in notification was accompanied by a decrease of treatment completion rates, from 74% in the previous analysis 11 to 66% in the present study. This probably also reflects a improvement in the IL-TB, with more loss to follow-up classified correctly. Again, proportion of treatment completion was heterogeneous across cities. Besides differences in local pandemic response, a possible explanation is the differential uptake of the 3HP regimen. The analysis of the factors associated to treatment outcomes was, however, out of the scope of our study.

Overall, these findings suggest that amid the public health emergency, there might have been insufficient workforce to complete all healthcare tasks, with absenteeism of healthcare workers and reallocation of human resources ^13^. Notifications may have been considered a less important task during the pandemic, with recovery when the pandemic was controlled.

Our study presents a few limitations. The data source is a recently established notification system. Notification of TB preventive treatment is not mandatory, although drug provision to the health facility depends on notification, which forces pharmacies to notify the cases. Brazil is a large country, and only 35% of the database was analyzed. Moreover, the decision to allocate missing outcomes to ongoing treatment or loss to follow-up is arbitrary, and new updates may show a recovery on treatment completion rates. In addition, the progressive uptake of the 3HP regimen could result in future increased completion rates. Finally, the database does not allow for evaluating losses in the previous steps of the TB infection cascade.

Despite these limitations, this study shows an encouraging rapid recovery of TB preventive treatment prescriptions after the initial phase of the pandemic. Our findings may be an incentive for policy makers, healthcare professionals, and clients to continue expanding TB preventive treatment and ensuring treatment completion. We recommend the dissemination of information about the importance of TB preventive treatment in reducing TB, as well as the allocation of resources to support this initiative. However, more prompt notification of prescription and outcomes is needed to allow for more agile surveillance and interventions.

With the recent establishment of the Brazilian Interministerial Committee for the Elimination of Tuberculosis and Other Socially Determined Diseases ^15^, we believe that TB prevention should be one of the most rewarding tasks to be encouraged.
